# Cannabinoid receptor 2 plays a central role in renal tubular mitochondrial dysfunction and kidney ageing

**DOI:** 10.1111/jcmm.16857

**Published:** 2021-08-19

**Authors:** Shan Zhou, Xian Ling, Ping Meng, Ye Liang, Kunyu Shen, Qinyu Wu, Yunfang Zhang, Qiyan Chen, Shuangqin Chen, Youhua Liu, Lili Zhou

**Affiliations:** ^1^ State Key Laboratory of Organ Failure Research National Clinical Research Center of Kidney Disease Division of Nephrology Nanfang Hospital Southern Medical University Guangzhou China; ^2^ Department of Central Laboratory Huadu District People’s Hospital Southern Medical University Guangzhou China; ^3^ Department of Nephrology the First People's Hospital of Foshan Foshan China

**Keywords:** CB2, kidney ageing, mitochondrial dysfunction, tubular cell, β‐catenin

## Abstract

Kidney is one of the most important organs in maintaining the normal life activities. With the high abundance of mitochondria, renal tubular cell plays the vital role in functioning in the reabsorption and secretion of kidney. Reports have shown that mitochondrial dysfunction is of great importance to renal tubular cell senescence and subsequent kidney ageing. However, the underlying mechanisms are not elucidated. Cannabinoid receptor 2 is one of the two receptors responsible for the activation of endocannabinoid system. CB2 is primarily upregulated in renal tubular cells in chronic kidney diseases and mediates fibrogenesis. However, the role of CB2 in tubular mitochondrial dysfunction and kidney ageing has not been clarified. In this study, we found that CB2 was upregulated in kidneys in 24‐month‐old mice and d‐galactose (d‐gal)‐induced accelerated ageing mice, accompanied by the decrease in mitochondrial mass. Furthermore, gene deletion of CB2 in d‐gal‐treated mice could greatly inhibit the activation of β‐catenin signalling and restore the mitochondrial integrity and Adenosine triphosphate (ATP) production. In CB2 knockout mice, renal tubular cell senescence and kidney fibrosis were also significantly inhibited. CB2 overexpression or activation by the agonist AM1241 could sufficiently induce the decrease in PGC‐1α and a variety of mitochondria‐related proteins and trigger cellular senescence in cultured human renal proximal tubular cells. CB2‐activated mitochondrial dysfunction and cellular senescence could be blocked by ICG‐001, a blocker for β‐catenin signalling. These results show CB2 plays a central role in renal tubular mitochondrial dysfunction and kidney ageing. The intrinsic mechanism may be related to its activation in β‐catenin signalling.

## 1 INTRODUCTION

Kidney is responsible for the vital physiological activities such as excretion, filtration, reabsorption and secretion in body.^1^ Renal tubular cell is fundamental to maintain the normal renal function.^2^ To meet the extraordinary needs of energy, through its respiratory chain complexes, high abundance of mitochondria in renal tubular cells conduct oxidative phosphorylation to produce ATP.^3^ Hence, the abnormal mitochondria in renal tubular cells would certainly lead to the damage of the metabolic state and also greatly affect the normal renal function. Large reports have shown that mitochondrial dysfunction is highly correlated with the decline of renal function.^4^, ^5^ In the multiple aetiologies, ageing is undoubtedly the very important mediator of mitochondrial dysfunction and the subsequent cell senescence in renal tubular cells^6^ and decline of renal function.^7^ In aged group, the excessive loss of mitochondrial mass^6^, ^8^ is accompanied by the high morbidity of kidney diseases.^9^ To mitigate mitochondrial reactive oxygen species (ROS) production not only protects against mitochondrial dysfunction and tubular cell senescence, but also retards age‐related renal fibrosis,^6^ suggesting the central state of mitochondrial quality control in kidney ageing. However, the underlying mechanisms of mitochondrial dysfunction in renal tubular cells are not clearly determined.

Cannabinoid receptors belong to G‐protein coupled seven‐span transmembrane receptor (GPCR), including CB1 and CB2.^10^ As the receptors in endocannabinoid system, they are highly related to immunity, development, memory, metabolism and other important physiological processes.^11^, ^12^, ^13^ The most recent studies show that cannabinoid receptors also participate in the pathogenesis of many diseases such as cardiovascular diseases, neurodegenerative diseases and especially kidney diseases.^1^, ^14^, ^15^, ^16^ Both CB1 and CB2 were found to be important mediators in the progression of renal fibrosis.^15^, ^17^ In our recent publication, CB2 was proved to induce the fibrotic change in renal tubular cells through β‐catenin‐activated pathway.^15^ Notably, β‐catenin activation is a very important mediating factor in renal tubular cell senescence and mitochondrial dysfunction, and age‐related renal fibrosis.^6^ However, the role of CB2 in the mitochondrial dysfunction of renal tubular cells has not been delineated. Moreover, the relationship between CB2 and tubular cell senescence should also be clarified.

In this study, we examined the expression of CB2 in aged kidneys and identified the mediative role of CB2 in the accelerated ageing. We also proved that CB2 is an important player in promoting mitochondrial dysfunction in tubular cells. Our study provides a new clue for the pathogenic role of endocannabinoid system in kidney health.

## 2 MATERIAL AND METHODS

### 2.1 *Animal* *models*

Male C57BL/6 mice, aged at 2‐month‐old and 24‐month‐old, were purchased from the Experimental Animal Center of Southern Medical University (Guangzhou, China). Six‐ to eight‐week‐old breeding pairs of the CB2^–/–^ (CB2 knockout) and CB2^+/+^ (wild‐type) male mice in C57BL/6N background were purchased from Cyagen Biosciences (stock no. KOCMP‐21582‐Cnr2; Cyagen Biosciences, Guangzhou, China) and bred in Experimental Animal Center of Southern Medical University under pathogen‐free conditions. Gene deletion of CB2 was confirmed by RT‐PCR. For the accelerated ageing model, unilateral nephrectomy was carried out in age‐matched CB2^+/+^ and CB2^–/–^ male mice on C57BL/6 background. All mice undergoing surgeries were treated with general anaesthesia. One week after surgery, the mice were administered subcutaneous injections of d‐gal (G0750; Sigma‐Aldrich) at 150mg/kg/day for 6 weeks. Kidney tissues were collected for various analyses. The detailed experimental designs were shown in Figure 3A. The animal experiments were approved by the Animal Ethic Committee at Southern Medical University.

### 2.2 *Cell* *culture and treatment*

Human proximal tubular epithelial cells (HKC‐8) were provided by Dr. Lorraine C. Racusen (Johns Hopkins University, Baltimore, MD, USA). No contamination was detected. HKC‐8 cells were transfected with empty vector (pcDNA3), CB2 overexpression vector (pCMV‐CB2) using Lipofectamine 2000 transfection reagent (11668019, Thermo) or followed by the stimulation of ICG‐001 at 10μM. HKC‐8 cells were treated with D‐gal at 10mg/ml (G0750; Sigma‐Aldrich), AM1241 (ab120934; Abcam) at 10 μM, or pretreated with XL‐001 at 10 μM for 1 h. Whole‐cell lysates were prepared and subjected to Western blot analyses or ATP detection. The detailed experimental designs and dosages were shown in figure legends respectively.

### 2.3 *Western* *blot analysis*

Proteins are separated on the basis of size by Western blot analyses. Briefly, the cultured tubular cells and kidney tissues were collected and ground in lysis buffer and their protein concentrations were measured using BCA protein concentration determination. The homogenates were heated at 100℃ for 10 minutes to fully denature the protein and then subjected to SDS‐PAGE electrophoresis. After electrophoresis, the proteins were transferred to a PVDF (polyvinylidene fluoride) membrane (Merck Millipore), blocked in blocking buffer (5% bovine serum albumin or skim milk) for 1 h and then incubated with primary antibodies overnight at 4°C and a secondary horseradish peroxidase‐conjugated antibody for 1 h at room temperature. The antigen‐antibody complexes were visualized using an ECL kit (Applygen). The primary antibodies used were as follows: anti‐CB2 (ab45942; Abcam), anti‐fibronectin (F3648; Sigma), anti‐α‐SMA (a2547; Sigma‐Aldrich), anti‐α‐SMA (ab5694; Abcam), anti‐β‐catenin (610154; BD Biosciences), anti‐Klotho (AF1819; R&D Systems), anti‐p14 ^ARF^ (ab185620; Abcam), anti‐p16^INK4A^ (ab189034; Abcam), anti‐p19^ARF^ (ab202225; Abcam), anti‐γH2AX (ab26350; Abcam), anti‐PGC‐1α (ab54481; Abcam), anti‐Cytb (SAB1304939; Sigma‐Aldrich), anti‐COX1 (SAB1301619; Sigma‐Aldrich), anti‐COX2 (sc23984, Santa Cruz), anti‐TFAM (PB0413; Boster), anti‐TOMM20 (ab186735; Abcam), anti‐α‐tubulin (RM2007; Ray Antibody Biotech), anti‐GAPDH (RM2002; Ray Antibody Biotech) and anti‐β‐actin (RM001; Ray Antibody Biotech).

### 2.4 *Reversetranscription and real*‐*time PCR*

Total RNA was obtained using TRIzol RNA isolation system (Life Technologies, Grand Island, NY) according to the manufacturer's instruction. Reverse transcription (RT) PCR was performed using HiScript III RT SuperMix for qPCR (R323‐01, Vazyme, China).

The first strand of complementary DNA was synthesized using 1 μg of RNA in 20 μl of reaction buffer containing 4 x gDNA wiper mix and 5× HiScript III qRT SuperMix.

Real‐time PCR was performed on ABI PRISM 7000 Sequence Detection System (Applied Biosystems, Foster City, CA) and using ChamQTMSYBR®qPCR Master Mix (High ROX Premixed) (Q341‐02/03, Vazyme, China). After initial denaturing at 95℃ for 30 seconds, the amplification protocol consisted of 40 cycles of denaturing at 95℃ for 10 seconds, annealing and extension at 60℃ for 30 seconds. The RNA levels of various genes were calculated after normalized by β‐actin. The sequences of the primer pairs used in PCR are shown in Table S1.

### 2.5 *Histology*, *immunohistochemical staining*

Paraffin‐embedded (3 μm thickness) kidney sections were prepared in a routine procedure. Periodic acid‐Schiff (PAS), and Sirius red staining (DC0040, Leagene Biotechnology, Beijing, China) were performed according to the manufacturer's instruction. Immunohistochemical staining was performed using routine protocol. Slides were incubated with antibodies against anti‐Klotho (AF1819; R&D Systems), anti‐fibronectin (F3648; Sigma), anti‐γH2AX (ab2893; Abcam), anti‐PGC‐1α (ab54481; Abcam), anti‐TOMM20 (ab186735; Abcam), Biotin‐sp‐conjugated affinipure donkey anti‐mouse, anti‐rabbit IgG or anti‐goat IgG (Jackson Immuno‐Research Laboratories, West Grove, PA). Images were taken by a microscope DP 27 CCD camera (Olympus, Japan).

### 2.6 *Immunofluorescence* *staining*

Kidney cryosections were fixed with 4% paraformaldehyde solution for 15 min at room temperature. Primary antibodies were as follows: anti‐active‐β‐catenin (4270s; Cell Signaling Technology), anti‐TOMM20 (ab186735; Abcam), anti‐Lotus Tetragonolobus Lectin (LTL) (FL‐1321; VECTOR Laboratories), anti‐Peanut Agglutinin (PNA)(FL‐1071; VECTOR Laboratories), anti‐Dolichos Biflorus Agglutinin (DBA)(FL1031; VECTOR Laboratories). After washing with TBS‐T, slides were incubated with Cy2 or Cy3‐conjugated donkey anti‐mouse or anti‐rabbit IgG (Jackson Immuno‐Research Laboratories, West Grove, PA). Nuclei were stained with DAPI (C1006, Beyotime) according to the manufacturer's instructions. Images were taken by confocal microscopy (Leica TCS SP2 AOBS, Leica Microsystems, Buffalo Grove, IL).

### 2.7 *In* *situ hybridization*

Kidney cryosections (3 μm) were fixed with 4% formaldehyde and incubated with CB2 probes labelled with digoxigenin at 40°C overnight. The expression of CB2 was assayed by fluorescence staining using a Fluorescence in situ hybridization kit (MK2530‐m; Boster technology). Nuclei were stained with DAPI (C1006, Beyotime) according to the manufacturer's instructions. Images were taken by confocal microscopy (Leica TCS SP2 AOBS, Leica Microsystems, Buffalo Grove, IL). CB2 probes: 5’‐ACCGCTACCTATGTCTGTGTTACCCGCCTACCTAC‐3’, 5’‐ACCAGGACAG GCAGGTGCCTGGGATAGCTCGGATG‐3’, and 5’‐AGCACTGCCTGATAGGCTGGAAGAAGTATCTACAG‐3’.

### 2.8 *Transmission* *electron microscopy*

Kidney cortex was collected and fixed in 1.25% glutaraldehyde/0.1 M phosphate buffer followed by resin embedding and ultrafine section making. Slides were subjected to assess kidney tubular mitochondrial ultrastructure under an electron microscope (JEOL JEM‐1010).

### 2.9 SA‐β‐gal and Adenosine triphosphate detection

Frozen sections (3 μm) and cultured cells were stained for SA‐β‐gal activity according to the manufacturer's instructions (#9860; Cell Signaling Technology). Kidney tissues or HKC‐8 cells were collected to be assessed by an adenosine triphosphate (ATP) Assay kit (S0027, Beyotime).

### 2.10 *Quantifications* *of staining*

Slides stained with Sirius red, immunohistochemical, immunofluorescence and SA‐β‐gal staining were observed at high‐powered (x400, x1000) fields from randomly selected fields. Each section contained 10 fields. Quantification of fibrotic lesions or positive staining was assessed by a computer‐aided technique (Image Pro Plus).

### 2.11 *Statistical* *analyses*

All of the data are expressed as the mean ± standard error of the mean (SEM). Statistical analysis was carried out using SPSS 20.0 (SPSS Inc, Chicago, IL). Comparisons were made by the Student t test or t’ test when comparing two groups or via one‐way analysis of variance (one‐way ANOVA) followed by the Least Significant Difference or Dunnett's T3 procedure for comparison of more than two groups. A value of *p *< 0.05 was considered to be statistically significant.

## 3 RESULTS

### 3.1 *CB2* *is upregulated in aged kidneys*

**FIGURE 1:**
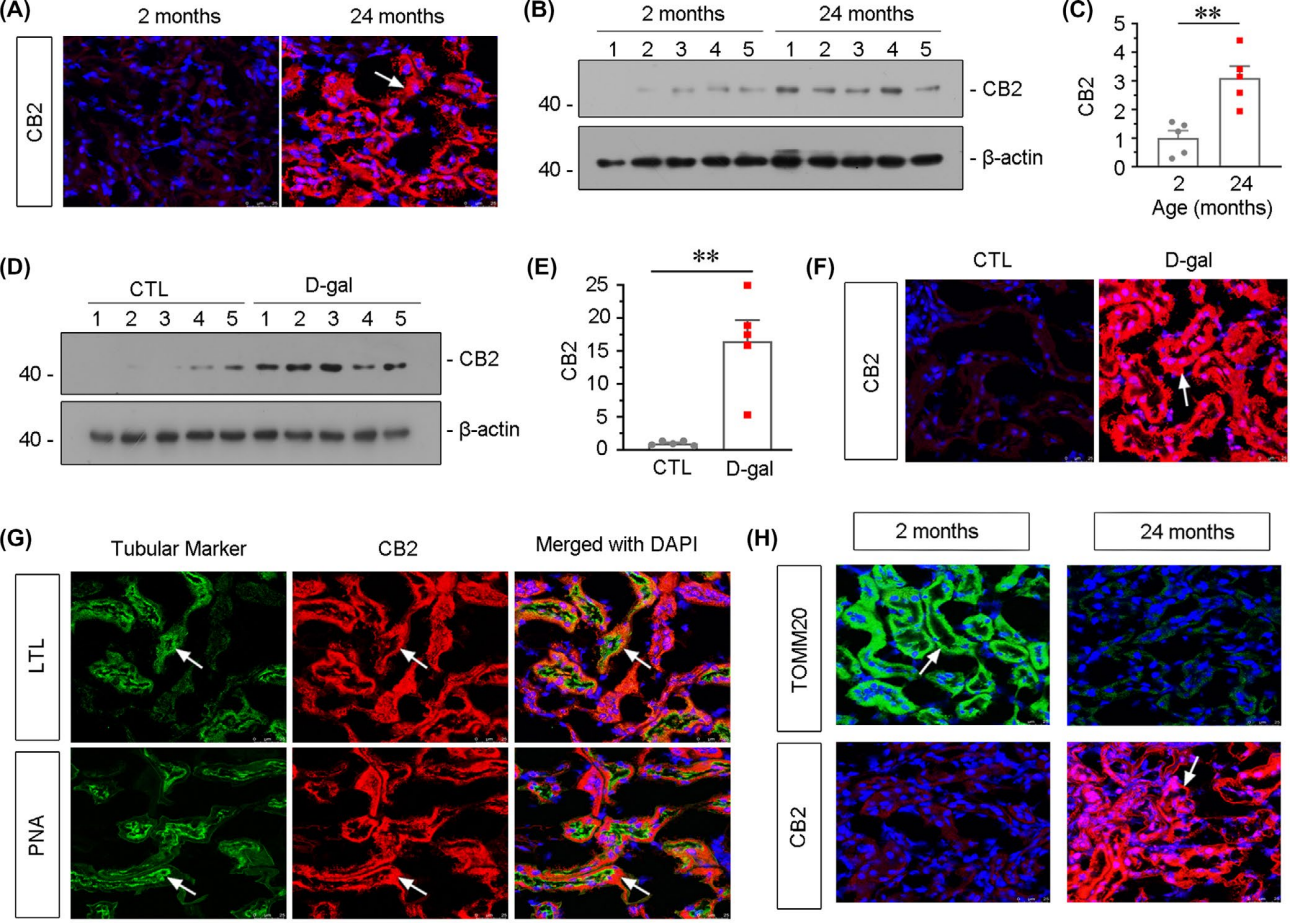
CB2 is upregulated in aged kidneys. (A) Representative micrographs showing CB2 expression in kidneys from 2‐month‐old and 24‐month‐old mice. Cryosections were subjected to fluorescence in situ hybridization (FISH) staining for CB2. Arrow indicates positive staining. scale bar, 25 μm. (B‐E) Representative Western blot and quantitative data showing renal expression of CB2 from 2‐month‐old and 24‐month‐old mice (B and C) or mice which were administered subcutaneous injections of d‐gal at 150mg/kg/day for 6 weeks (D and E). Numbers (1–5) indicate each individual animal in a given group. **p < 0.01 versus 2‐month‐old mice group or the sham control group (n = 5). (F) Representative images showing renal expression of CB2 in d‐gal‐treated mice. Cryosections were subjected to fluorescence in situ hybridization (FISH) staining for CB2. Arrow indicates positive staining. scale bar, 25 μm. (G) Representative micrographs showing the colocalization of CB2 and various segment‐specific tubular markers in kidneys. Frozen kidney sections were stained for CB2 (red) using FISH and various segment‐specific tubular markers (green) by immunofluorescence. The following segment‐specific tubular markers were used: proximal tubule, lotus tetragonolobus lectin (LTL); distal tubule, peanut agglutinin (PNA); arrows indicate positive tubules with colocalization of CB2 and specific tubular markers. Scale bar, 25 μm. (H) Representative micrographs showing the expression of CB2 and TOMM20 in tubules in 2‐month‐old and 24‐month‐old mice. Cryosections were subjected to FISH staining of CB2 (red) and stained with TOMM20 (green) antibody by immunofluorescence. Arrows indicate positive staining. Scale bar, 25μm

To investigate the role of CB2 in ageing, we assessed the expression of CB2 in the kidneys from naturally aged mice. The expression of CB2 was first tested by fluorescence in situ hybridization (FISH). As shown in Figure 1A, CB2 was highly upregulated in renal tubules in 24‐month‐old mice. The similar result was also observed when CB2 was tested by Western blotting (Figure 1B,C). We next assessed the expression of CB2 in d‐gal‐treated mice, an accelerated ageing model.^6^, ^18^ As shown in Figure 1D–F, CB2 expression was also greatly increased and primarily upregulated in renal tubules. To identify the location of CB2 in renal tubules, the co‐staining for CB2 with different segment‐specific tubular cell markers was performed in the kidneys in 24‐month‐old mice. As shown in Figure 1G, CB2 was co‐localized with lotus tetragonolobus lectin (LTL), a marker of proximal tubules, and peanut agglutinin (PNA), a marker of distal tubules. While CB2 is barely co‐stained with dolichos biflorus agglutinin (DBA), a marker of collecting ducts (data not shown). To assess the correlation between CB2 and mitochondrial function, the expression fashion of translocase of outer mitochondrial membrane 20 (TOMM20, a mitochondrial marker) and CB2 was assessed by immunofluorescence and FISH, respectively. As shown in Figure 1H, compared with the high expression of TOMM20 in the kidneys in 2‐month‐old mice, TOMM20 expression was dramatically decreased in the aged kidneys in 24‐month‐old mice. Interestingly, CB2 expression presented an opposite fashion (Figure 1H), suggesting that CB2 plays a critical role in ageing and is related to mitochondrial dysfunction.

### 3.2 ***CB2*** ***gene ablation does not affect kidney ageing or mitochondrial function in young mice***

**FIGURE 2:**
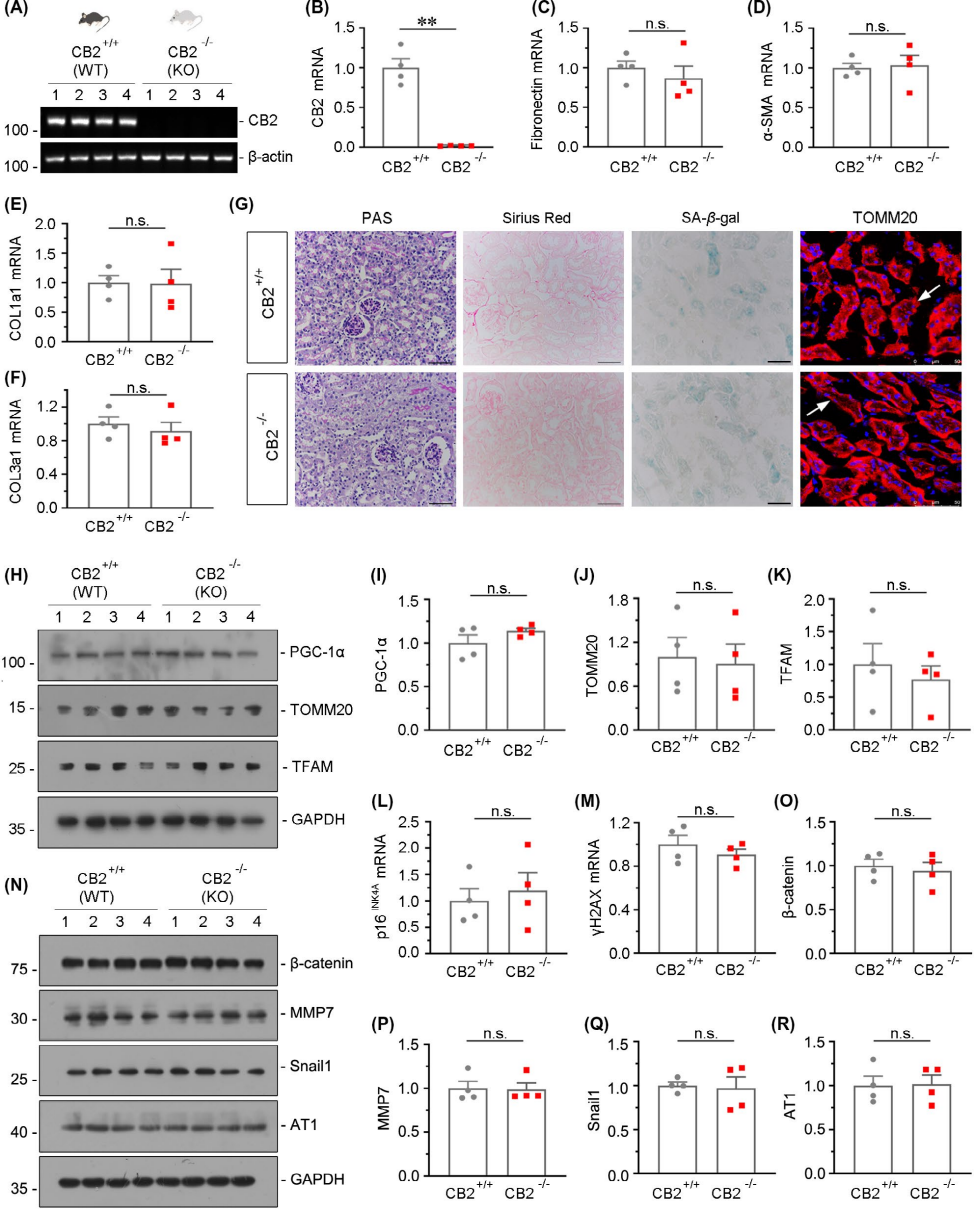
CB2 gene ablation does not affect kidney ageing or mitochondrial function in young mice. (A) RT‐PCR analyses showing renal expression of CB2 in wild‐type mice (WT) and CB2 knockout mice (KO). Numbers (1–4) indicate each individual animal in a given group. (B‐F) Quantitative real‐time PCR results showing renal expression of CB2, fibronectin, α‐SMA, CollagenⅠa1 and CollagenⅢa1 in WT and KO mice. **p < 0.01, n.s. versus WT mice group (n = 4). n.s.: no significance. (G) Representative micrographs showing Periodic acid‐Schiff (PAS) staining, Sirius red staining, senescence‐associated β‐galactosidase activity (SA‐β‐gal) staining and the expression of TOMM20. Paraffin‐embedded kidney sections were subjected to PAS and Sirius red staining. Frozen kidney sections were stained for SA‐β‐gal activity and TOMM20. Scale bar, 50 μm. (H‐K) Representative Western blot and quantitative data showing renal expression of PGC‐1α, TOMM20 and TFAM in WT and KO mice. Numbers (1–4) indicate each individual animal in a given group. n.s. versus WT mice group (n=4). n.s.: no significance. (L‐M) Quantitative real‐time PCR results showing renal expression of p16^INK4A^ and γH2AX in WT and KO mice. n.s. versus WT mice group (n = 4). n.s.: no significance. (N‐R) Representative Western blot and quantitative data showing renal expression of β‐catenin, MMP7, Snail1 and AT1 in WT and KO mice. Numbers (1–4) indicate each individual animal in a given group. n.s. versus WT mice group (n = 4). n.s.: no significance

The gene ablation efficiency of CB2 was first testified by RT‐PCR and quantitative real‐time PCR (Figure 2A,B). We next analysed the expression of the fibrosis‐related genes fibronectin, α‐smooth muscle actin (α‐SMA), collagen type 1 alpha 1 (COL1a1) and collagen type 3 alpha 1 (COL3a1). As shown in Figure 2C–F, there is no difference of these genes in wild‐type and CB2 knockout mice. Furthermore, the results of Periodic acid‐Schiff (PAS) and Sirius red staining also showed that there was no obvious change in tubular damage and fibrotic injury after CB2 gene ablation (Figure 2G). We next tested the senescence in two groups of mice. As shown in Figure 2G, L and M, the results of the senescence‐associated β‐galactosidase (SA‐β‐gal) staining, and analysis of mRNA expression of p16^INK4A^ and γH2AX, showed that CB2 gene ablation did not affect kidney ageing. We next assessed mitochondrial function. The protein expression of peroxisome proliferator‐activated receptor‐C coactivator‐1α (PGC‐1α), TOMM20 and mitochondrial transcription factor A (TFAM), the mitochondrial biogenesis‐related transcription factors, was analysed by Western blotting. As shown in Figure 2H–K, they were also not changed by CB2 gene ablation. We also tested the expression of β‐catenin and its signalling‐related proteins, such as MMP7, Snail1 and AT1. There were no changes in these mice after CB2 gene deficiency (Figure 2N–R).

### 3.3 *β*‐*catenin* *signalling is inhibited by CB2 gene ablation in D*‐*gal*‐*treated mice*

**FIGURE 3:**
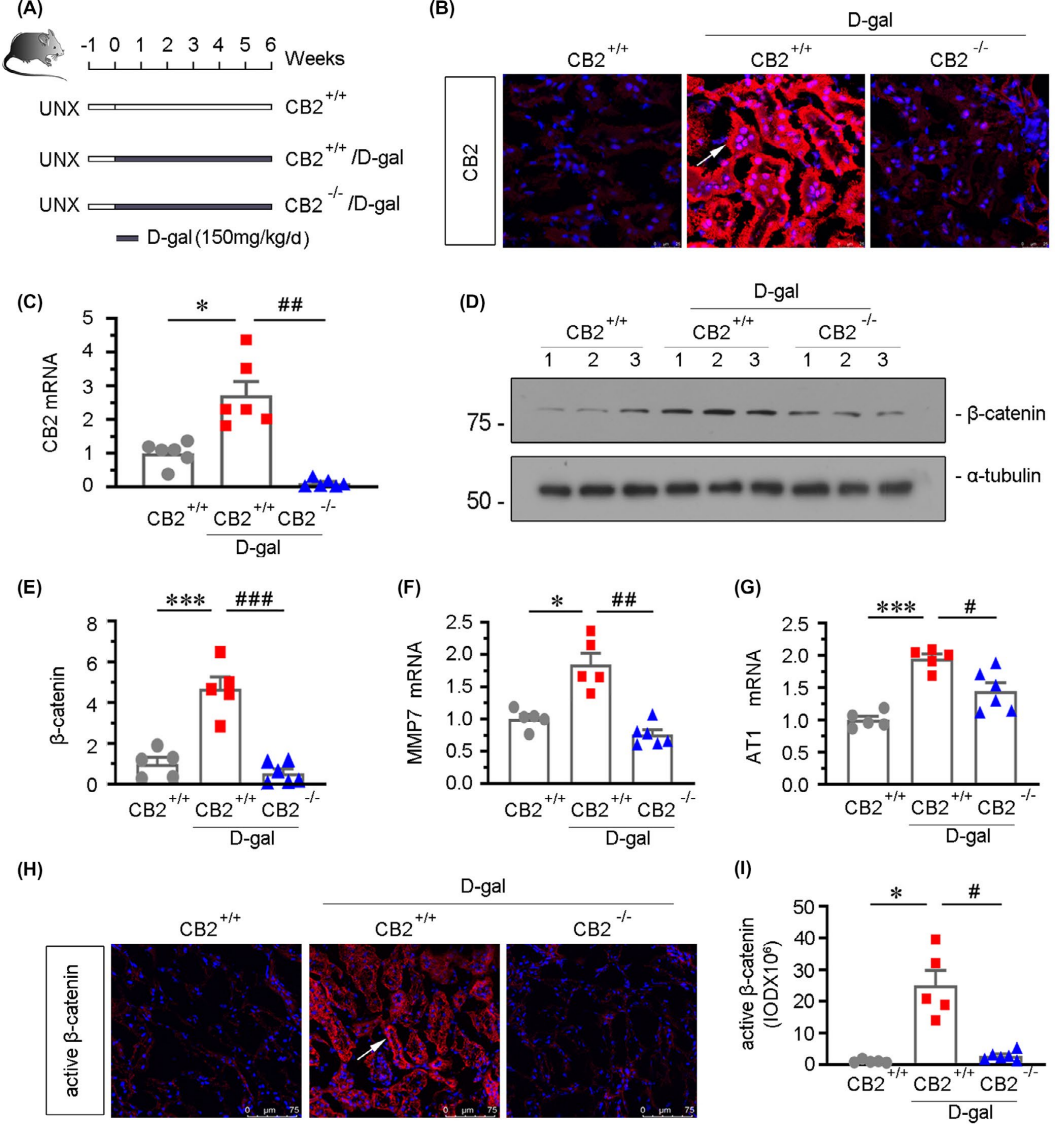
β‐catenin signalling is inhibited by CB2 gene ablation in d‐gal‐treated mice. (A) Experimental design. Black bar indicated that mice were administered subcutaneous injections of d‐gal at 150mg/kg/day for 6 weeks after surgery for 1 week. UNX: unilateral nephrectomy. (B) Representative micrographs showing renal expression of CB2 in different groups. Cryosections were subjected to fluorescence in situ hybridization (FISH) staining for CB2. Arrow indicates positive staining. scale bar, 25 μm. (C) Quantitative real‐time PCR results showing renal expression of CB2. *p < 0.05 versus WT mice group alone; ^##^ *p *< 0.01 versus the d‐gal‐treated WT mice group alone (n = 5–6). (D and E) Representative Western blot and quantitative data showing renal expression of β‐catenin. Numbers (1–3) indicate each individual animal in a given group. ***p < 0.001 versus WT mice group alone; ^###^ *p *< 0.001 versus the d‐gal‐treated WT mice group alone (n = 5–6). (F and G) Quantitative real‐time PCR results showing renal expression of MMP7 and AT1. *p < 0.05 and ***p < 0.001 versus WT mice group alone; ^#^ *p *< 0.05 and ^##^ *p *< 0.01 versus the d‐gal‐treated WT mice group alone (n = 5–6). (H) Representative micrographs showing the expression of active β‐catenin. Frozen kidney sections were stained with an antibody against active β‐catenin. Arrow indicates positive staining. Scale bar, 75μm. (I) Quantitative data showing quantification of positive staining. *p < 0.05 versus WT mice group alone; ^#^ *p *< 0.05 versus the d‐gal‐treated WT mice group alone (n = 5–6)

To investigate the potential role of CB2 in kidney ageing and mitochondrial dysfunction, we adopted an accelerated ageing mouse model.^6^, ^18^ The wild‐type and CB2 knockout mice were first performed unilateral nephrectomy and then injected with d‐gal at 150mg/kg/day for 6 weeks (Figure 3A). We first tested the expression of CB2 by FISH and qRT‐PCR. As shown in Figure 3B,C, although administration of d‐gal induced the upregulation of CB2 in tubules, CB2 expression was nearly completely inhibited in d‐gal‐treated CB2 knockout (CB2^−/−^) mice. As β‐catenin signalling is an important mediator of kidney ageing,^6^ we next analysed the expression of β‐catenin and its downstream targets MMP7 and AT1.^19^, ^20^ As shown in Figure 3D, E, H and I, d‐gal treatment induced the upregulation of β‐catenin and its activation, and however, gene ablation of CB2 largely blocked these changes. The similar results were observed when MMP7 and AT1 expression levels were assessed by qRT‐PCR (Figure 3F,G). These findings suggest that CB2 could regulate kidney ageing through activating β‐catenin signalling.

### 3.4 *CB2* *deficiency protects renal mitochondrial homeostasis in the accelerated ageing mice*

**FIGURE 4:**
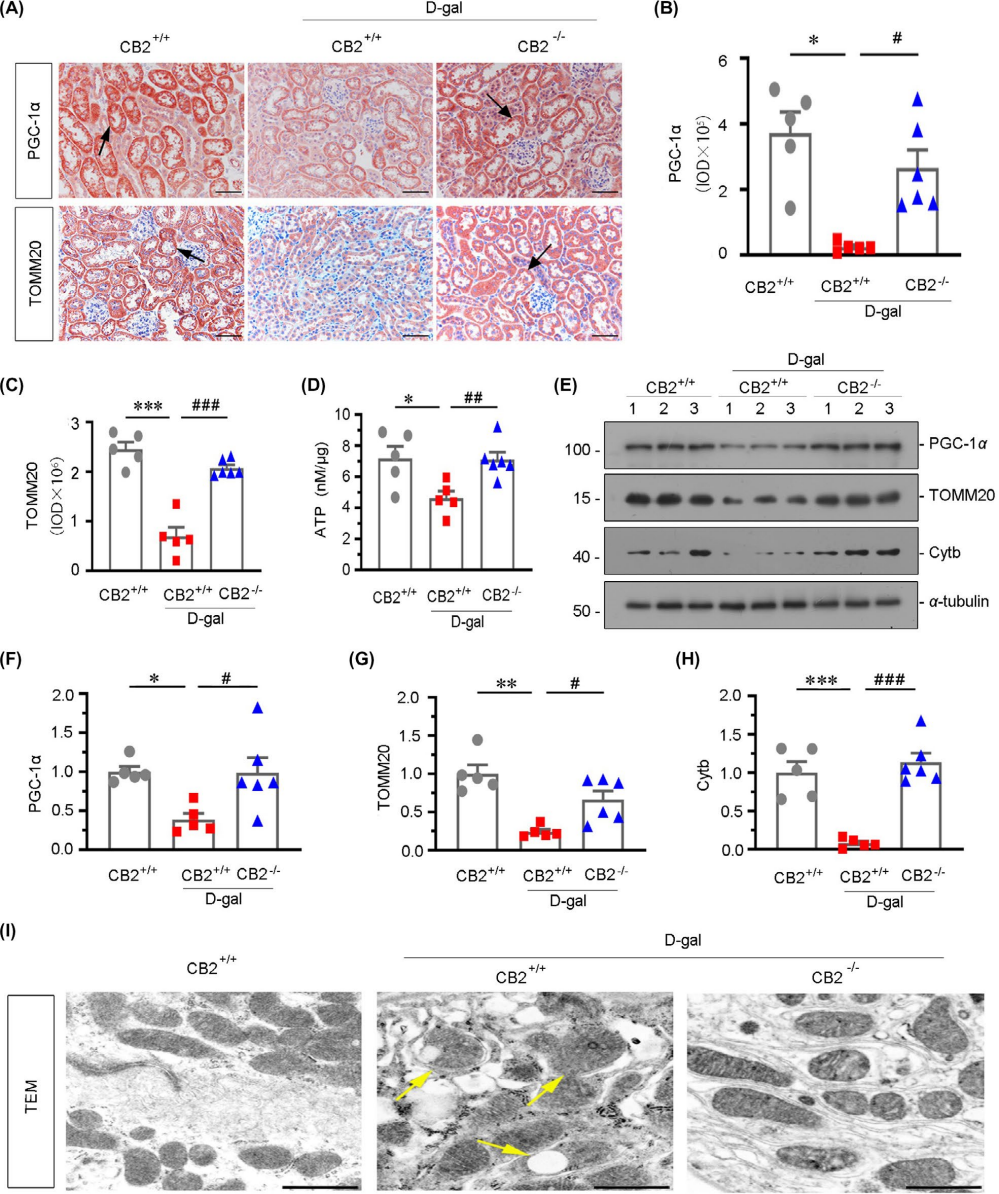
CB2 deficiency protects renal mitochondrial homeostasis in the accelerated ageing mice. (A) Representative micrographs showing renal expression of PGC‐1α and TOMM20 in different groups. Paraffin‐embedded kidney sections were immunostained with an antibody against PGC‐1α or TOMM20. Arrows indicate positive staining. Scale bar, 50 μm. (B‐C) Quantitative data showing quantification of positive staining. *p < 0.05, ***p < 0.001 versus WT mice group alone; ^#^ *p *< 0.05, ^###^ *p *< 0.001 versus the d‐gal‐treated WT mice group alone (n = 5–6). (D) Representative graph showing the production of adenosine triphosphate (ATP) in different groups. *p < 0.05 versus WT mice group alone; ^##^ *p *< 0.01 versus the d‐gal‐treated WT mice group alone (n = 5–6). (E–H) Representative Western blot and quantitative data showing renal expression of PGC‐1α, TOMM20 and Cytb. Numbers (1–3) indicate each individual animal in a given group. *p < 0.05, **p < 0.01, ***p < 0.001 versus the WT mice group alone; ^#^ *p *< 0.05, ^###^ *p *< 0.001 versus the d‐gal‐treated WT mice group alone (n = 5–6). (I) Representative transmission electron microscopy graphs showing mitochondrial ultrastructure of renal tubular cells in different groups. Arrows indicate damaged and abnormal‐shaped mitochondria. Scale bar, 1μm

To identify the role of CB2 in mitochondrial dysfunction, we first investigated the expression of peroxisome proliferator‐activated receptor gamma coactivator‐1 alpha (PGC‐1α), a key factor modulating mitochondrial biogenesis, and TOMM20 by immunostaining. As shown in Figure 4A–C, d‐gal treatment triggered the downregulation of PGC‐1α and TOMM20, while gene ablation of CB2 significantly restored their expression. Similar results were observed when PGC‐1α and TOMM20 were assessed by Western blotting analyses (Figure 4E–G). Furthermore, the expression of cytochrome b (Cytb), an mtDNA‐encoded OXPHOS complex III subunit, was also restored by CB2 gene deletion in d‐gal‐treated mice (Figure 4E and H). To further assess the role of CB2 in mitochondrial dysfunction, we analysed the production of adenosine triphosphate (ATP). As shown in Figure 4D, administration of d‐gal decreases the production of ATP, and however, the gene ablation of CB2 could significantly promote the increase in ATP levels. The mitochondrial ultrastructure was also inspected by transmission electron microscopy (TEM). As shown in Figure 4I, compared with the normal rod‐shape and well‐arranged mitochondria in control mice, d‐gal treatment induced the breakdown of mitochondrial ultrastructure, leading to the most mitochondria to be swelling, fragmented and disorganized, while CB2 gene knockout largely preserved the normal structure of mitochondria. These results show that CB2 plays a central role in renal tubular mitochondrial dysfunction.

### 3.5 ***CB2*** ***gene ablation ameliorates kidney ageing***

**FIGURE 5:**
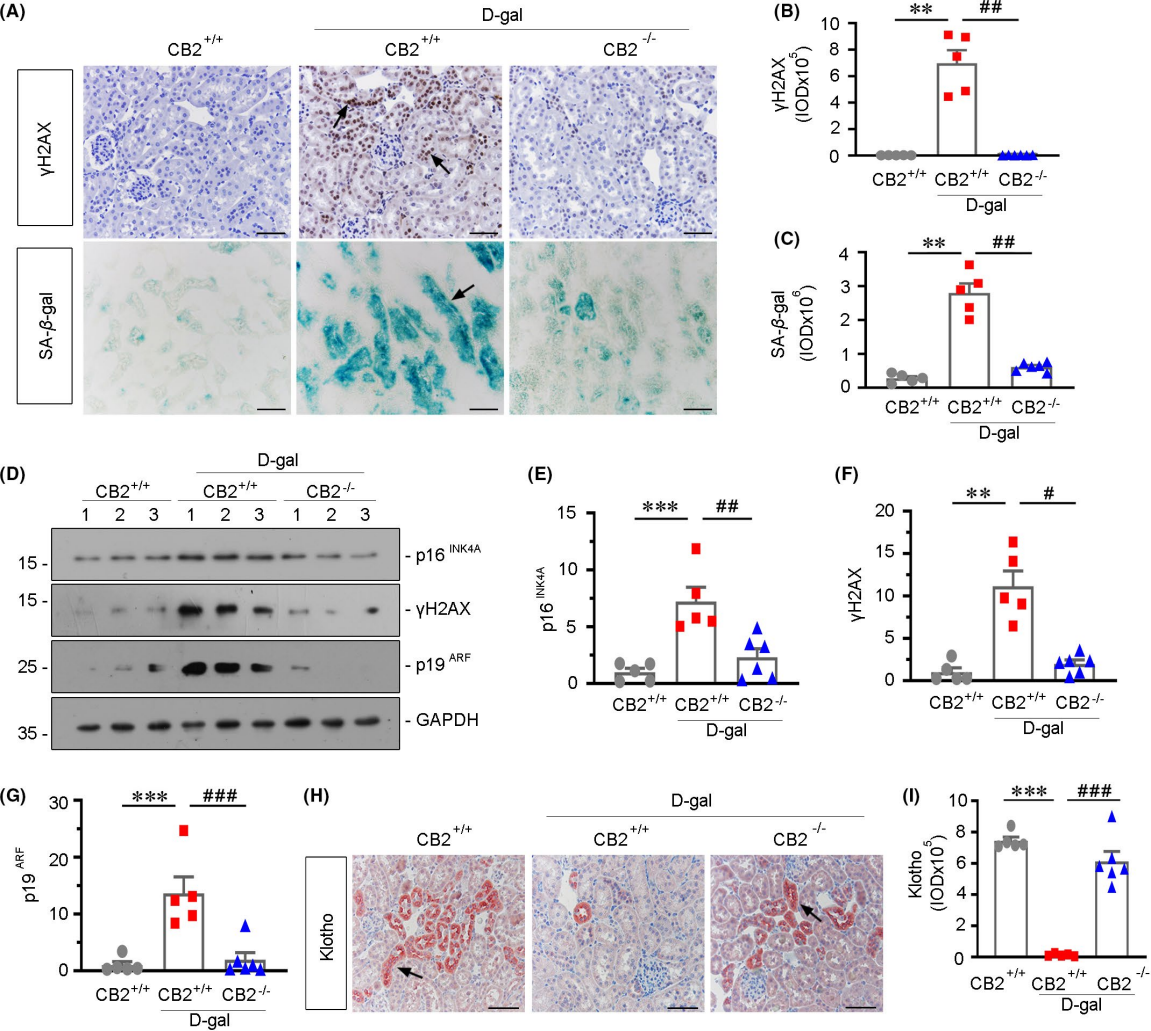
CB2 gene ablation ameliorates kidney ageing. (A) Representative micrographs showing renal expression of γH2AX and SA‐β‐gal activity in different groups. Paraffin‐embedded kidney sections were immunostained with an antibody against γH2AX (top). Frozen kidney sections were stained for SA‐β‐gal activity (bottom). Arrows indicate positive staining. Scale bar, 50 μm. (B‐C) Quantitative data showing quantification of positive staining. **p < 0.01 versus WT mice group alone; ^##^ *p *< 0.01 versus the d‐gal‐treated WT mice group alone (n = 5–6). (D–G) Representative Western blot and quantitative data showing renal expression of p16^INK4A^, γH2AX and p19^ARF^ in different groups. Numbers (1–3) indicate each individual animal in a given group. **p < 0.01, ***p < 0.001 versus the WT mice group alone; ^#^ *p *< 0.05, ^##^ *p *< 0.01, ^###^ *p *< 0.001 versus the d‐gal‐treated WT mice group alone (n = 5–6). (H and I) Representative micrographs showing renal expression of klotho in different groups (H). Paraffin‐embedded kidney sections were immunostained with an antibody against klotho. Arrows indicate positive staining. Scale bar, 50 μm. (I) Quantitative data showing quantification of positive staining. ***p < 0.001 versus the WT mice group alone; ^###^ *p *< 0.001 versus the d‐gal‐treated WT mice group alone (n = 5–6)

We further assessed the role of CB2 in kidney ageing. As shown in Figure 5A,B d‐gal treatment strongly induced the upregulation of γH2AX, a marker for DNA double‐strand breaks and an important senescence marker, while it was nearly completely inhibited in CB2 knockout mice. We next assessed the cellular senescence by SA‐β‐gal staining. The senescent tubules, which appeared the bright‐blue granular staining, were greatly induced by d‐gal administration, but evidently inhibited by gene ablation of CB2, as shown in Figure 5A and C. Similar results were observed when γH2AX, p16^INK4A^ and p19^ARF^, the important factors triggering senescent pathway, were assessed by Western blotting (Figure 5D–G). Klotho is an anti‐ageing protein,^21^ which is a protector for mitochondrial function in renal tubular cells.^5^ We next assessed the expression of Klotho. As shown in Figure 5H and I, CB2 gene ablation could also largely preserve the expression of Klotho.

### 3.6 *CB2deficiency retards age*‐*related kidney fibrosis*

**FIGURE 6:**
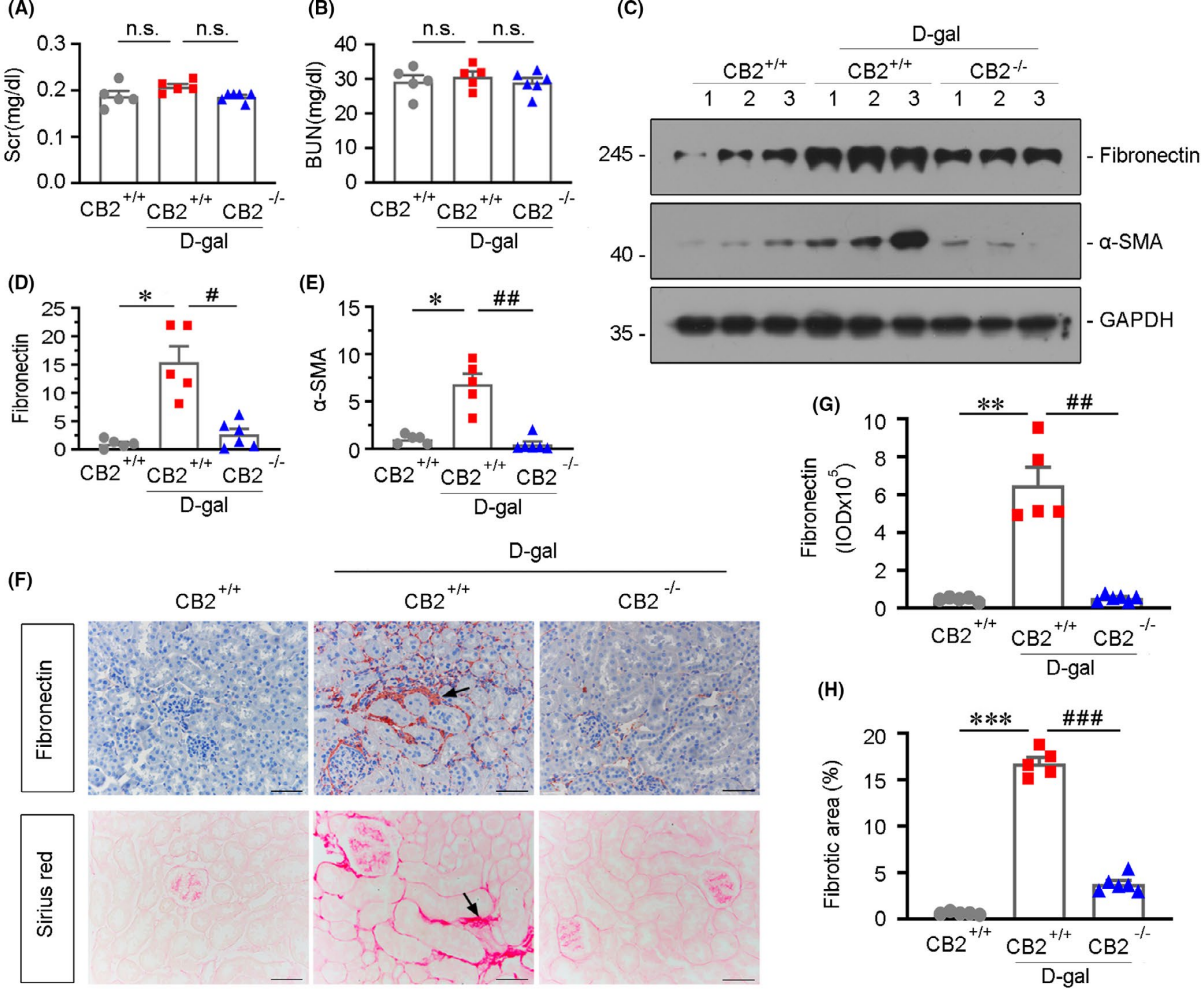
CB2 deficiency retards age‐related kidney fibrosis. (A and B) Quantitative data showing serum creatinine (Scr) and blood urea nitrogen (BUN) levels in different groups. n.s.: no significance. (C–E) Representative Western blot and quantitative data showing renal expression of fibronectin and α‐SMA in different groups. Numbers (1–3) indicate each individual animal in a given group. *p < 0.05 versus the WT mice group alone; ^#^ *p *< 0.05, ^##^ *p *< 0.01 versus the d‐gal‐treated WT mice group alone (n = 5–6). (F–H) Representative micrographs showing renal expression of fibronectin and Sirius red staining in different groups. Paraffin‐embedded kidney sections were stained with Sirius red and were immunostained with an antibody against fibronectin. Arrows indicate positive staining. Scale bar, 50 μm. Quantitative data showing quantification of positive staining of fibronectin (G) and fibrotic area (H). **p < 0.01, ***p < 0.001 versus the WT mice group alone; ^##^ *p *< 0.01, ^###^ *p *< 0.001versus the d‐gal‐treated WT mice group alone (n = 5–6)

Furthermore, we tested serum creatinine (Scr) and blood urea nitrogen (BUN) levels in mice injected with d‐gal, but there were no significant changes among three groups (Figure 6A,B). We then tested renal fibrosis. As shown in Figure 6C–E d‐gal treatment induced the upregulation of fibronectin and α‐SMA, the two fibrosis markers, while they were largely blocked in CB2 knockout mice. The similar result was observed when fibronectin was assessed by immunostaining (Figure 6F,G). Furthermore, the fibrotic lesions were also analysed by Sirius red staining. As shown in Figure 6F and H, the quantification of fibrotic lesions further confirmed the protective role of CB2 gene deficiency in age‐related kidney fibrosis.

### 3.7 *CB2* *induces mitochondrial dysfunction and cellular senescence in vitro*

**FIGURE 7:**
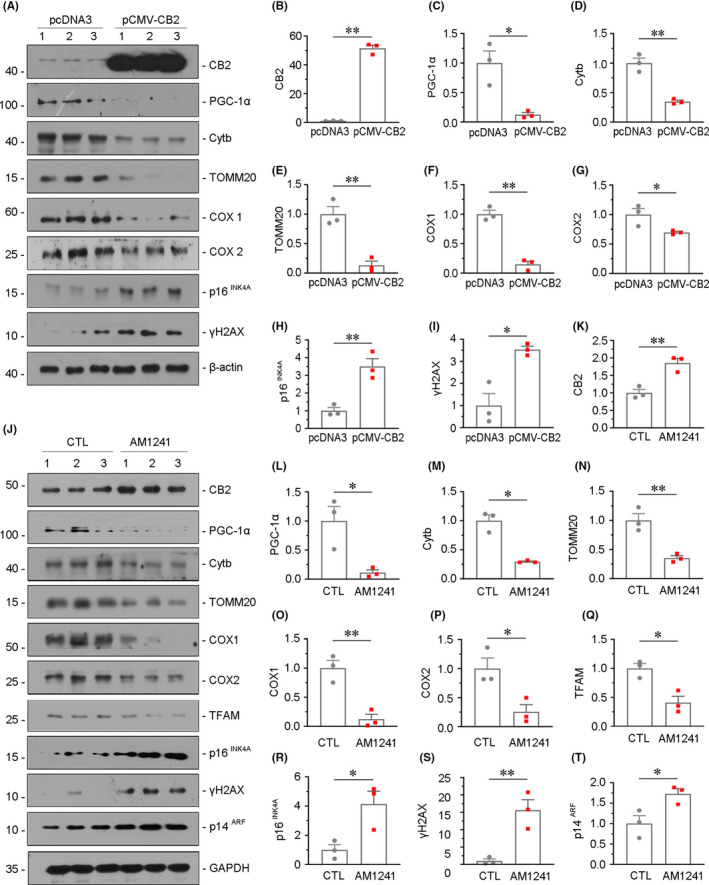
CB2 induces mitochondrial dysfunction and cellular senescence in vitro. (A–I) Representative Western blot and quantitative data showing the expression of CB2, PGC‐1α, Cytb, TOMM20, COX1, COX2, p16^INK4A^, γH2AX in HKC‐8 cells. HKC‐8 cells were transfected with CB2 expression plasmid (pCMV‐CB2) for 24 h. *p < 0.05, **p < 0.01 versus the pcDNA3 group (n = 3). (J–T) Representative Western blot and quantitative data showing the expression of CB2, PGC‐1α, Cytb, TOMM20, COX1, COX2, TFAM, p16^INK4A^, γH2AX and p14^ARF^ in HKC‐8 cells. HKC‐8 cells were treated with AM1241 (10 μM) for 48h. *p < 0.05, **p < 0.01 versus the control group (n = 3)

We then assessed the role of CB2 in tubular cell senescence in vitro. The human proximal tubular epithelial cell line (HKC‐8) was cultured and transfected with CB2 expression plasmid. The expression of CB2 was first testified by Western blotting (Figure 7A,B). We next assessed the expression of mitochondria‐related proteins. The key transcription factor of mitochondria biogenesis, PGC‐1α, was greatly decreased by CB2 overexpression (Figure 7A and C). In addition, transfection with CB2 expression plasmid induced the downregulation of mitochondrial OXPHOS subunits Cytb, TOMM20, cytochrome c oxidase 1 (COX1) and cytochrome c oxidase 2 (COX2) (Figure 7A,D–G). Furthermore, CB2 overexpression triggered the expression of p16^INK4A^ and γH2AX, the two senescence markers (Figure 7A, H and I).

HKC‐8 cells were also treated with aminoalkylindole (AM) 1241, a CB2‐specific agonist (Figure 7J and K). Consistent with CB2 gene overexpression, AM1241 treatment downregulated the expression of PGC‐1α, Cytb, TOMM20, COX1, COX2 and TFAM (Figure 7J,L–Q), suggesting the role of CB2 in mitochondrial dysfunction. Furthermore, AM1241 treatment also increased the expression of p16^INK4A^, γH2AX and p14^ARF^ (Figure 7J,R–T), the three senescence markers.

### 3.8 *β*‐*catenin* *plays a mediative role in CB2*‐*induced mitochondrial dysfunction and cellular senescence*

**FIGURE 8:**
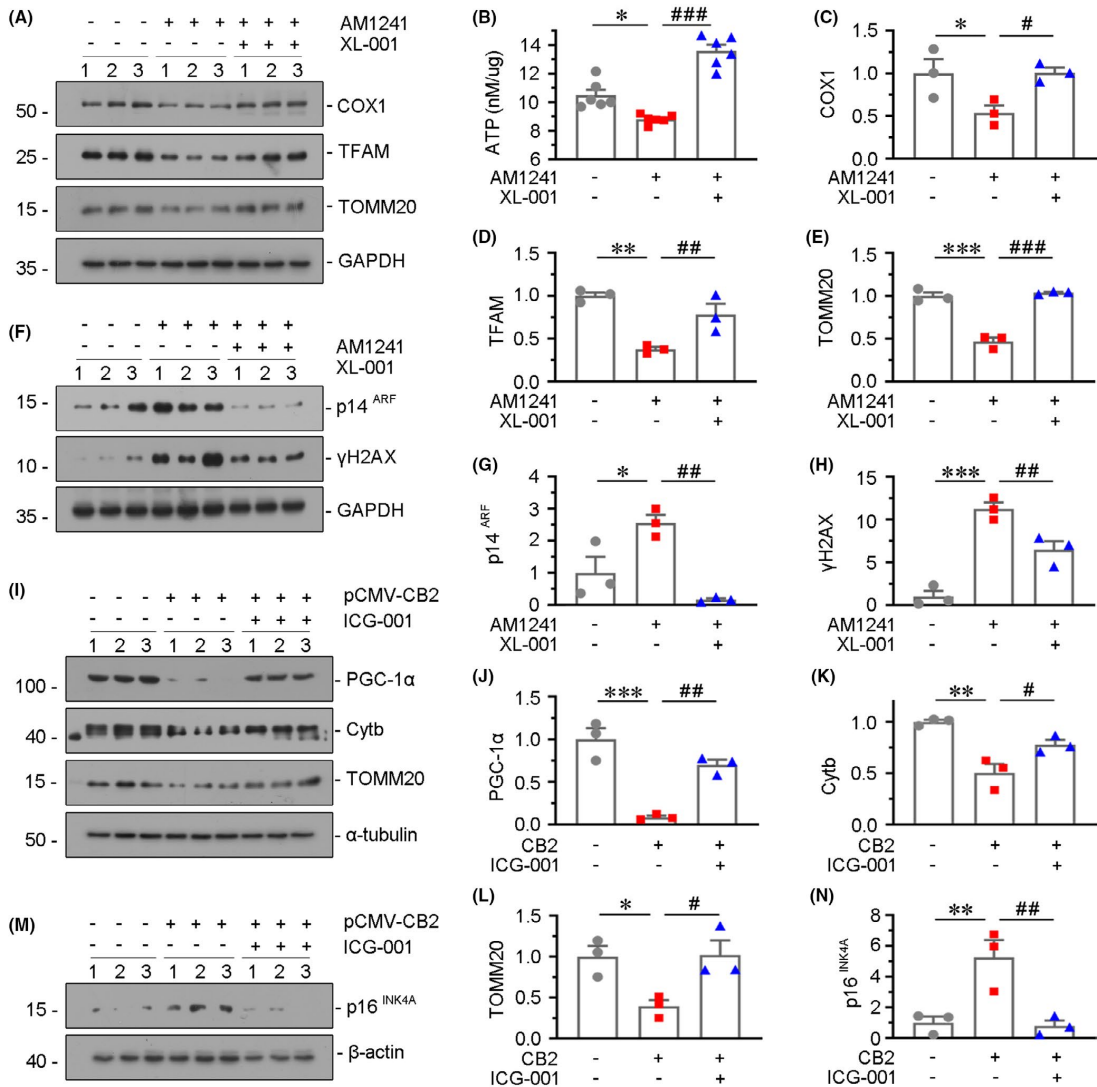
β‐catenin plays a mediative role in CB2‐induced mitochondrial dysfunction and cellular senescence. (A–E) Representative Western blot (A, F) and quantitative data (C–E, G and H) showing the expression of COX1, TFAM, TOMM20, p14^ARF^ and γH2AX in HKC‐8 cells. HKC‐8 cells were treated with AM1241 (10 μM) for 48 h and pretreated with XL‐001 (10 μM) for 1 h. Quantitative data graph (B) showing the production of adenosine triphosphate (ATP) in HKC‐8 cells. *p < 0.05, **p < 0.01, ***p < 0.001 versus the control group alone; ^#^ *p *< 0.05, ^##^ *p *< 0.01, ^###^ *p *< 0.001versus the AM1241 group alone (n = 3). (I–N) Representative Western blot (I, M) and quantitative data (J–L, N) showing the expression of PGC‐1α, Cytb, TOMM20 and p16^INK4A^ in HKC‐8 cells. HKC‐8 cells were transfected with CB2 expression plasmid (pCMV‐CB2), followed by the stimulation of ICG‐001 at 10μM for 24 h *p < 0.05, **p < 0.01, ***p < 0.001 versus the control group alone; ^#^ *p* < 0.05, ^##^ *p* < 0.01versus the pCMV‐CB2 group alone (n = 3)

We further assessed the role of CB2 in kidney ageing using XL‐001, a novel inverse agonist of CB2 invented by our group.^14^ As shown in Figure 8A–H, pretreatment with XL‐001 could largely inhibited AM1241‐induced mitochondrial dysfunction and cellular senescence.

In our recent study, we found β‐catenin plays a vital role in kidney ageing and renal tubular cell senescence^6^, ^22^ and mediates CB2‐induced GPCR signal wave,^15^ and we then tested its role in CB2‐induced kidney ageing. HKC‐8 cells were pretreated with ICG‐001, a small molecule inhibiting β‐catenin‐mediated gene transcription, and then transfected with CB2 expression plasmid. As shown in Figure 8I–L, ICG‐001 pretreatment largely inhibited CB2‐induced decrease in PGC‐1α, Cytb and TOMM20. In addition, the expression of p16^INK4A^ was also tested. As shown in Figure 8M,N, CB2‐induced increase in p16^INK4A^ was greatly blocked by ICG‐001 pretreatment.

### 3.9 *CB2* *plays a central role in the accelerated ageing in renal tubular cells*

**FIGURE 9:**
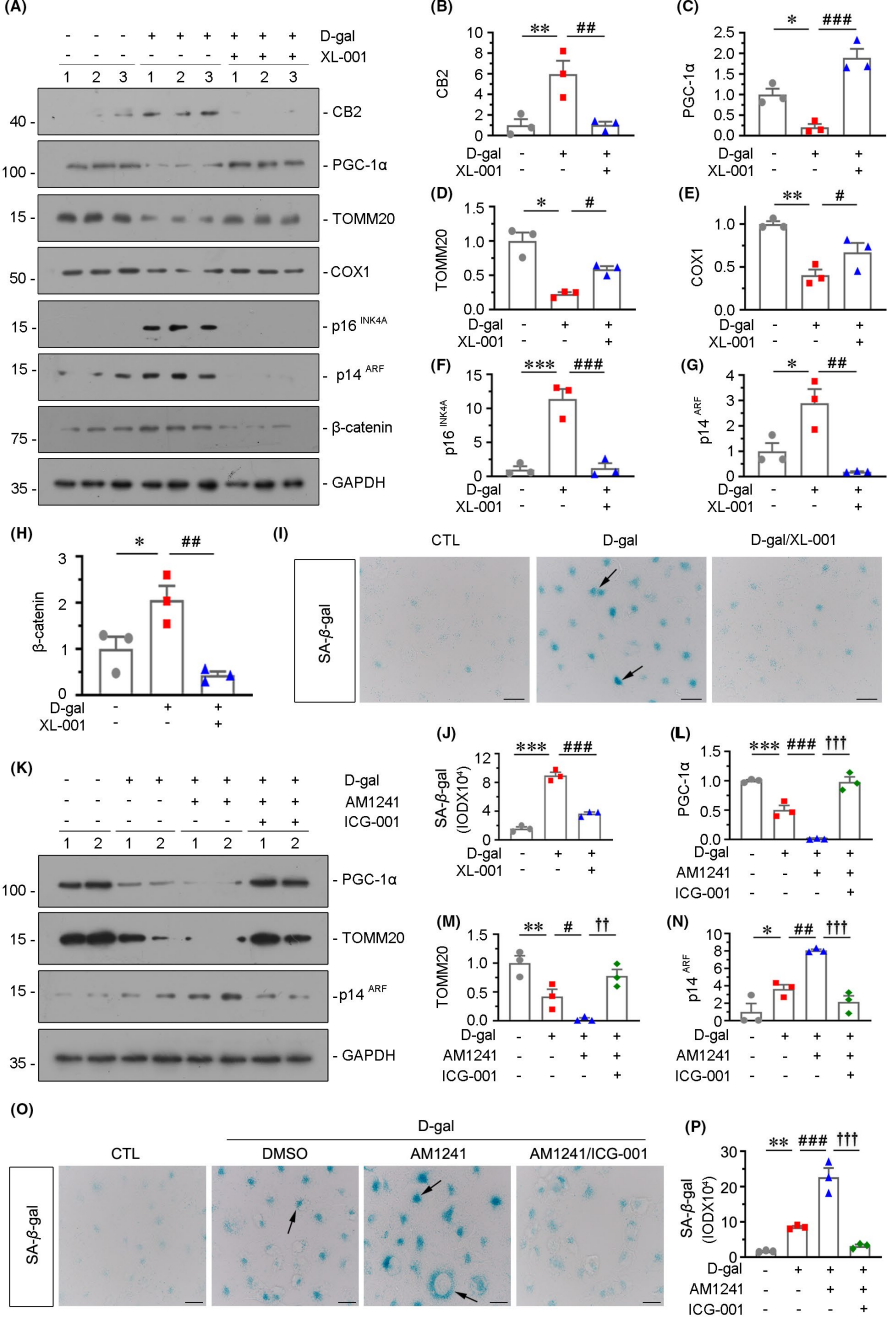
CB2 plays a central role in the accelerated ageing in renal tubular cells. (A–H) Representative Western blot and quantitative data showing the expression of CB2, PGC‐1α, TOMM20, COX1, p16^INK4A^, p14^ARF^ and β‐catenin in HKC‐8 cells. HKC‐8 cells were treated with D‐gal at 10mg/ml for 72h and pretreated with XL‐001 (10μM) for 1 h. *p < 0.05, **p < 0.01, ***p < 0.001 versus the control group alone;^#^ *p* < 0.05, ^##^ *p* < 0.01, ^###^ *p* < 0.001 versus the d‐gal group alone (n = 3). (I and J) Representative micrographs and quantitative data showing SA‐β‐gal activity in different groups. Frozen kidney sections were stained for SA‐β‐gal activity. Arrows indicate positive staining. Scale bar, 20 μm. ***p < 0.001 versus the control group alone; ^###^ *p* < 0.001 versus the d‐gal group alone (n = 3). (K–N) Representative Western blot and quantitative data showing renal expression of PGC‐1α, TOMM20 and p14^ARF^ in HKC‐8 cells. HKC‐8 cells were treated with D‐gal at 10mg/ml or cotreated with AM1241 (10 μM) for 72 h and pretreated with ICG‐001 (10 μM) for 1 h. *p < 0.05, **p < 0.01, ***p < 0.001 versus the control group alone; ^#^ *p* < 0.05, ^##^ *p* < 0.01, ^###^ *p* < 0.001 versus the d‐gal group alone; ^††^ *p* < 0.01, ^†††^ *p* < 0.001 versus the d‐gal+AM1241 group alone (n = 3). (O and P) Representative micrographs and quantitative data showing SA‐β‐gal activity in different groups. Frozen kidney sections were stained for SA‐β‐gal activity. Arrows indicate positive staining. Scale bar, 20 μm. **p < 0.01 versus the control group alone; ^###^ *p* < 0.001 versus the d‐gal group alone; ^†††^ *p* < 0.001 versus the d‐gal+AM1241 group alone (n = 3)

HKC‐8 cells were pretreated with XL‐001 and then treated with d‐gal. As shown in Figure 9A,B, XL‐001 inhibited the expression of CB2 in d‐gal treated cells. The mitochondrial function, cellular senescence and β‐catenin pathway were then assessed. As shown in Figure 9A,C–E, d‐gal treatment induced the decrease in PGC‐1α, TOMM20 and COX1, while pretreatment with XL‐001 largely restored their expression. Furthermore, the expression of p16^INK4A^ and p14^ARF^, the two senescence markers, was upregulated by d‐gal administration but significantly reduced by XL‐001 (Figure 9A and F–G). Incubation with d‐gal triggered an increase in SA‐β‐gal activity. Coculture with XL‐001 inhibited d‐gal‐induced cellular senescence (Figure 9I,J). Interesting, we have observed the downregulation of β‐catenin by XL‐001 in d‐gal‐treated cells (Figure 9A and H), suggesting that CB2 induces kidney ageing through activating β‐catenin pathway.

To further identify the role of β‐catenin in CB2‐induced kidney ageing, HKC‐8 cells were incubated with d‐gal and with AM1241 and cotreated with ICG‐001. As shown in Figure 9K–M, AM1241 further decreased d‐gal‐downregulated PGC‐1α and TOMM20 expression, but this was significantly blocked by co‐treatment with ICG‐001. Furthermore, d‐gal‐induced increase in p14^ARF^ was further elevated by AM1241, and however, co‐treatment with ICG‐001 could greatly inhibit this effect (Figure 9K and N). We observed the similar results in cultured cells. SA‐β‐gal activity staining showed that D‐gal and AM1241 aggravated tubular cell senescence in d‐gal‐treated cells, but treatment with ICG‐001 largely inhibited it (Figure 9O,P). These results further clarify that CB2 plays a central role in kidney ageing and is related with activation of β‐catenin.

## 4 DISCUSSION

The population ageing is becoming a big problem to the economic and health care society in the whole world.^23^ In the future 30 years, the aged group of population would occupy up to one‐fifth of the population.^24^ Ageing is one of the strongest inducer in the decline of organ function.^25^, ^26^ However, the inducing factors of ageing have not been demonstrated in detail. Among the multiple aetiologies of ageing, mitochondrial dysfunction is thought to be the very important mediator in acceleration of ageing and decline of organ function.^6^, ^27^

Mitochondrion is a very important organelle in eukaryotic cells. With a two‐layer membrane coated, the negative voltage difference between the outer and inner layer facilitates the transmission of metabolites such as acetone into tricarboxylic acid cycle.^28^ Furthermore, the oxidative respiratory chain in the inner layer of mitochondrion carries out a chain reaction to produce energy through the electron transportation.^29^ As an organ with vigorous metabolism, kidney parenchymal cells, especially renal tubular cells, possess the high abundance of mitochondria to produce ATP for executing the reabsorption and excretion function.^30^ Renal tubular cell mitochondrial dysfunction is highly related to the damaged cell function.^31^ Our previous reports show that to protect against mitochondrial dysfunction could inhibit cellular senescence and age‐related fibrosis in kidneys.^6^ In aged organs, the damaged mitochondria are characterized with loss of mitochondrial mass, ROS production, fragmentation^6^ and abnormal calcium influx,^32^ etc., which further lead to the reduced production of ATP to form a vicious cycle. Therefore, mitochondrial quality control has been thought to stand at the centre of anti‐ageing strategies, while the underlying mechanisms of mitochondrial dysfunction in kidney ageing have not been elucidated.

Wnt/β‐catenin signalling is a conserved development pathway,^33^ and however, it is reactive in multiple kinds of kidney diseases.^34^ Interestingly, we have found that it is also overactivated in aged kidneys.^6^ The activation of β‐catenin is accompanied by the loss of mitochondria mass and overproduced ROS, suggesting the highly intimate correlation of β‐catenin with mitochondrial dysfunction. β‐catenin could be activated by Wnt‐triggered inhibition on its degradation^35^ and also could be stimulated by GPCR‐induced signal wave.^15^, ^36^, ^37^

Interestingly, we have found several GPCRs such CXCR4 and CB2 induce the kidney injury depending on β‐catenin activation.^15^, ^36^ CB2 was recently found to be primarily upregulated in the proximal and distal tubules in renal fibrosis model and promoted kidney fibrosis by orchestrating β‐catenin signalling, as reported by our recent work.^14^, ^15^ Notably, our recent findings show the accelerated cellular senescence in tubular cells plays the vital role in the progression of renal fibrosis.^22^ This suggests there would be a high correlation between CB2 and tubular cell ageing. However, this has not been experimentally proved.

In this study, we have verified the internal correlation between CB2 and kidney ageing. We first assessed the expression of CB2 in the kidneys in naturally ageing mice and d‐gal‐accelerated ageing mice. We found that CB2 is highly increased in aged kidneys and correlated with the decrease in mitochondrial mass (Figure 1). We next performed the study in CB2 knockout mice (Figure 2). The results show that gene ablation of CB2 could inhibit β‐catenin signalling (Figure 3), preserve mitochondrial function (Figure 4), retard cellular senescence (Figure 5) and greatly ameliorate age‐related kidney fibrosis (Figure 6). The induction of CB2 on tubular cell ageing and mitochondrial dysfunction was also proved in cultured proximal tubular cell line (Figure 7). Notably, ICG‐001, a small molecular compound which inhibits β‐catenin activation, could greatly block CB2‐induced cell ageing and mitochondrial damage (Figure 8). Furthermore, the high correlation between tubular cell ageing and mitochondrial dysfunction with CB2/β‐catenin pathway was also testified in d‐gal‐treated tubular cell culture (Figure 9). All of these results reveal the central role of CB2 in renal tubular mitochondrial dysfunction and kidney ageing.

CB2 belongs to GPCR and was previously thought to be responsible for immunity modulation.^38^ Its role in kidney diseases was recently proved by our group. Through the gene deletion of CB2, we found that CB2 plays a detrimental role in the progression of renal fibrosis^14^ and also clarified the internal interlaced relationship between CB2 and β‐catenin pathway.^15^ As β‐catenin pathway plays a vital role in kidney ageing and the promotion of mitochondrial dysfunction,^5^, ^6^ we hypothesize that CB2 could also act as an important mediator in kidney ageing. Through our findings, we conclude that the activation of endocannabinoid system, especially CB2, would undoubtedly affect the kidney function through an ageing‐promoted pathway. Considering the increasing use of exocannabinoid products for medial and other purposes,^39^ our studies provide an important health alert that the long‐term stimulation of this system would impose a heavy burden to the lifespan of organs.

## 5 CONCLUSION

We have uncovered CB2 is the important mediator in kidney ageing. The study, for the first time, identified CB2 plays a central role in kidney ageing and clarified CB2 could promote mitochondrial dysfunction in renal tubular cells and be related to β‐catenin activation. Given that the increased use of exocannabinoid products for medical and recreational purposes, our studies provide a warning that needs high attention that the long and heavy activation of CB2 receptor would accelerate kidney ageing and lead to the damage on the mitochondria, one of most important organelle in the body.

## CONFLICT OF INTEREST

The authors declared no competing interests.

## AUTHOR CONTRIBUTIONS

**Shan Zhou:** Data curation (equal); Methodology (lead); Writing‐original draft (equal); Writing‐review & editing (lead). **Xian Ling:** Data curation (lead); Methodology (equal); Writing‐original draft (lead); Writing‐review & editing (equal). **Ping Meng:** Data curation (equal); Formal analysis (lead); Investigation (lead); Methodology (equal). **Ye Liang:** Investigation (supporting); Methodology (supporting). **Kunyu Shen:** Data curation (supporting). **Qinyu Wu:** Methodology (supporting). **Yunfang Zhang:** Resources (supporting). **Qiyan Chen:** Resources (supporting). **Shuangqin Chen:** Investigation (supporting). **Youhua Liu:** Supervision (supporting). **Lili Zhou:** Conceptualization (lead); Supervision (lead).

## Supporting information

Table S1
