# Supplementary material for: Cannabinoid receptor 2 plays a central role in renal tubular mitochondrial dysfunction and kidney ageing
Source: J Cell Mol Med. 2021 Aug 19;25(18):8957–72. doi: 10.1111/jcmm.16857 (PMC8435409; doi:10.1111/jcmm.16857)
Supplement: Supplementary file 1 — Table S1 [file JCMM-25-8957-s001.docx]

**Supplementary Material**

**Table S1**

Nucleotide sequences of the primers used for RT-PCR or real-time PCR

| **Gene** | **Primer Sequence 5’ to 3’** | |
| --- | --- | --- |
|  | **Forward** | **Reverse** |
| CB2-Mouse | TATGCTGGTTCCCTGCACTG | GAGCGAATCTCTCCACTCCG |
| FN-Mouse | GATGAGCTTCCCCAACTGGT | CTGGGTTGTTGGTGGGATGT |
| α-SMA-Mouse | GAGGCACCACTGAACCCTAA | CATCTCCAGAGTCCAGCACA |
| COL1a1-Mouse | CTGGCGGTTCAGGTCCAAT | CTTGCCAGCTTCCCCATCA |
| COL3a1-Mouse | ATTGGGATGCAGCCACCTTG | ATGGGATCTCTGGGTTGGGG |
| P16^INK4A^-Mouse | CATCTGGAGCAGCATGGAGTC | GCACCGTAGTTGAGCAGAAGA |
| γH2AX-Mouse | GGTGCTCGAGTACCTCACTG | CTTGTTGAGCTCCTCGTCGT |
| MMP-7-Mouse | TAGGCGGAGATGCTCACTTT | TTCTGAATGCCTGCAATGTC |
| AT1-Mouse | CAGTTGGGAGGGACTGGATGA | GTTAAGTCCGGGAGAGCAGCA |
| β-actin-Mouse | cagctgagagggaaatcgtg | cgttgccaatagtgatgacc |
